# Fatal Superior Vena Cava Obstruction With High-Output Chylothorax in a Preterm Infant: A Complication of Central Venous Catheterization

**DOI:** 10.7759/cureus.71296

**Published:** 2024-10-12

**Authors:** Andreia D Constante, Daniel Virella, Maria J Lage, Fátima Pinto

**Affiliations:** 1 Pediatric Cardiology Department, Hospital de Santa Marta, Unidade Local de Saúde São José, Lisbon, PRT; 2 Neonatal Intensive Care Unit, Hospital de Dona Estefânia, Unidade Local de Saúde São José, Lisbon, PRT

**Keywords:** central venous catheter thrombosis, chylothorax, neonatal thrombosis, prematurity, superior vena cava obstruction

## Abstract

Chylothorax in the perinatal period may have congenital or acquired aetiologies. In premature infants, invasive procedures with thrombosis risk are common practice. We present a case of a 29-week gestation neonate, diagnosed on the 27th postnatal day with vegetation on the tip of the central venous catheter (CVC) and right auricle thrombosis, along with superior vena cava (SVC) syndrome, leading to significant bilateral chylothorax. Despite antithrombotic therapy, extensive intramural SVC obstruction persisted. Surgical intervention was considered high risk in such a preterm infant. Lung function declined progressively, and the neonate died from cardiorespiratory failure at the age of 2 months and 26 days. A post-mortem examination revealed minimal SVC lumen obstruction, emphasizing the potential lethality of secondary complications, regardless of successful thrombolysis. This case highlights both the life-threatening risk of neonatal SVC thrombosis associated with CVC and the need to assess the causes of chylothorax for coexisting aetiologies.

## Introduction

Chylothorax is the accumulation of chyle in the pleural cavity due to a leak in the lymphatic vessels and is the most common form of pleural effusion described in the perinatal period. Its composition is characterized by a mixture of fluids and chylomicrons, with a triglyceride level >110 mg/dL, a pleural fluid/serum triglyceride ratio >1.0, and a pleural fluid/serum cholesterol ratio <1.0 [1].

Secondary chylothorax, a rare neonatal disorder, results from dysfunction of the lymphatic system due to trauma, cardiothoracic surgery, obstruction, or raised pressure in the superior vena cava (SVC). Central venous catheters (CVC) are vital for managing critically ill infants, including preterm neonates, who undergo invasive procedures and face thrombosis risk. SVC thrombosis can lead to SVC syndrome and chylothorax, resistant to medical treatment, with high morbidity (30%) and mortality (18%) [2]. Surgical intervention is considered when medical therapy fails, but the optimal timing lacks consensus, complicated by age, vessel size, and vascular injury challenges in these critically ill patients.

## Case presentation

A 29-week premature female was transferred to our neonatal intensive care unit on her fifth postnatal day with the diagnosis of pneumoperitoneum. She underwent laparotomy, which revealed isolated ileal perforation. An ileostomy was placed, and a percutaneous 4Fr CVC was inserted in the left brachiocephalic trunk. The postoperative course was uneventful, but on the 27th postnatal day, she was diagnosed with a vegetation at the tip of the CVC, located in the right auricle, and patent ductus arteriosus (PDA). The CVC was removed; blood culture and culture of the tip were positive for *Staphylococcus capitis*. Antibiotics were started, along with intravenous paracetamol for PDA closure, and anticoagulation with low molecular weight heparin (LMWH) every 12 hours, adjusted to therapeutic anti-Xa factor levels. After ten days, a significant right pleural effusion required invasive ventilation and the first chest drain placement. Chylothorax was diagnosed through pleural fluid analysis, which revealed elevated triglycerides, among other findings (see Table 1). The condition evolved with increasing output rates (>16 mL/hour).

**Table 1:** Laboratory investigations of pleural fluid. LDH: lactate dehydrogenase.

Parameter	Result	Reference Range
Leukocytes	729/µL	0-500/µL
Polymorphonuclear cells	60/µL	0-300/µL
Mononuclear cells	669/µL	0-500/µL
Proteins	20.0 g/L	1.5-2.5 g/L
Glucose	71 mg/dL	60-80 mg/dL
LDH	277 U/L	100-300 U/L
Pancreatic amylase	<1 U/L	<1 U/L
Total cholesterol	41 mg/dL	30-60 mg/dL
Triglycerides	223 mg/dL	<100 mg/dL

The high output of fluid into the pleural space caused hypoalbuminemia, along with electrolyte and acid-base derangements, including hyponatremia and metabolic acidosis. To account for these losses, albumin and electrolytes were administered as needed. Renal, liver, and thyroid functions were monitored and remained within normal levels. Octreotide, parenteral nutrition, and a low-fat elemental formula diet were attempted for chylothorax control without significant clinical improvement.

**Figure 1:**
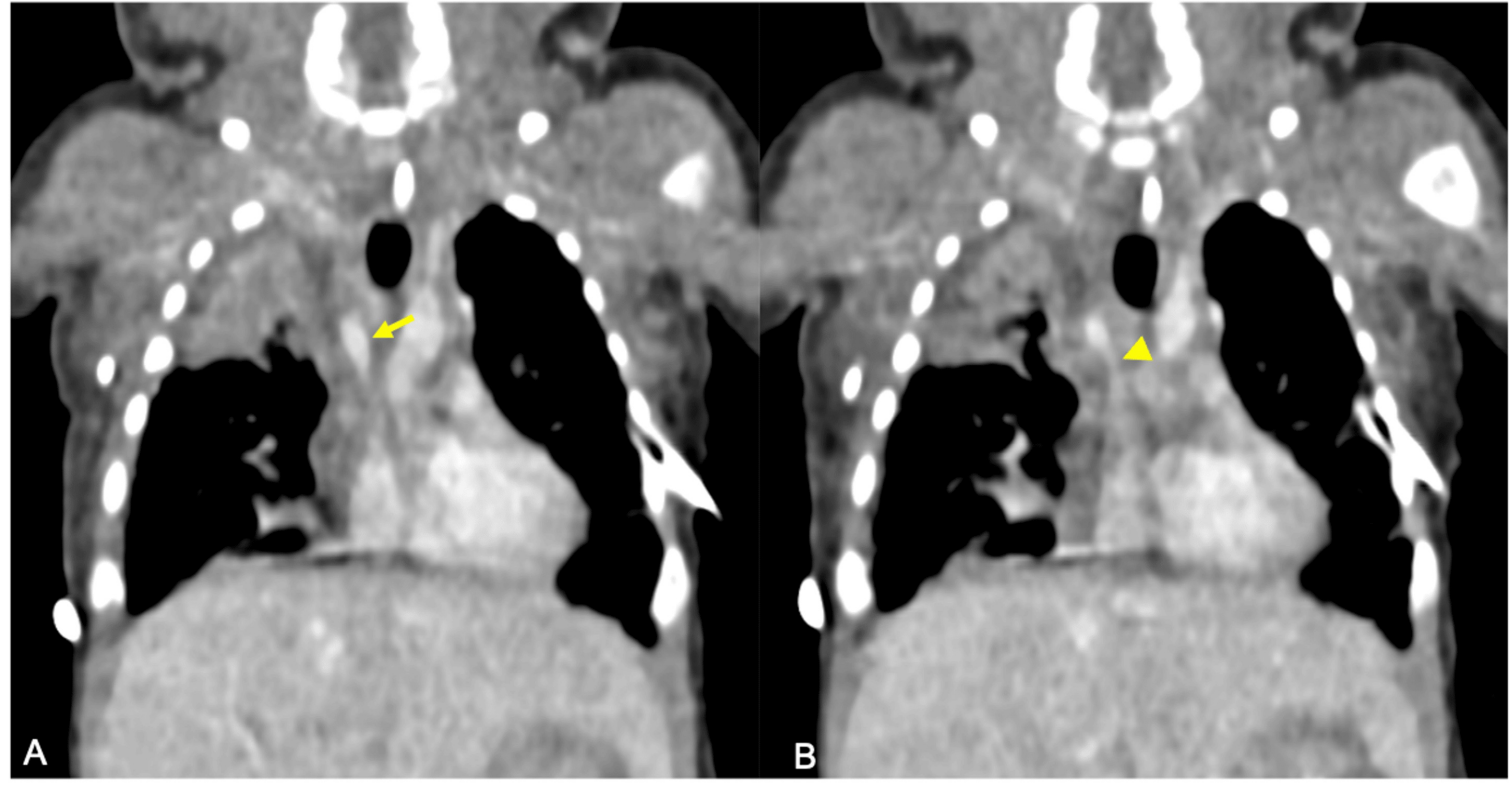
Coronal CT images illustrating the superior vena cava (SVC). Image A shows the normal caliber of the SVC (yellow arrow). Image B highlights the site of stenosis in the SVC (yellow arrowhead). Consolidation of the right upper lobe is also visualized.

Recurrent pneumothorax occurred bilaterally due to obstruction and externalization of the drainage catheter. After five days, there was no reduction in the thrombus or the severity of the obstruction, with a maintained gradient of 3 mmHg in the SVC. Anticoagulation was changed to a perfusion of unfractionated heparin (UH) (20 U/kg/h) as there were no known contraindications. Laboratory monitoring with frequent determinations of activated partial thromboplastin time (APTT) was challenging, with the potential risk of either subtherapeutic doses or bleeding. Due to persistent low antithrombin (AT) levels (around 10%), the LMWH anticoagulation regimen was resumed. Over time, the right auricle thrombus resolved, but turbulent flow persisted in the SVC. Clinical symptoms of SVC syndrome remained despite ongoing antithrombotic therapy. CT angiography confirmed the extensive intramural lesion in the SVC, obstructing venous return (Figure 1).

The hypothesis of surgical intervention presented a very high risk for a 1400 g preterm baby. Despite effective bilateral thoracic fluid drainage (200-300 mL daily), progressive lung function deterioration occurred, necessitating increasing ventilatory support, sedation, and analgesia, culminating in cardiorespiratory failure at two months and 26 postnatal days. The autopsy report was inconclusive; it excluded cardiac malformations and identified signs of respiratory infection and pulmonary hypertension. The thoracic duct was not visualized, and the superior vena cava lumen was patent.

## Discussion

We present a case of fatal chylothorax in a very preterm infant linked to SVC thrombosis from CVC placement. Chylothorax in infants and children can be congenital or acquired. Congenital factors include lymphatic malformations (e.g., lymphangiomatosis, lymphangiectasia, thoracic duct atresia) and syndromes (e.g., Down, Noonan, Turner). Acquired causes include trauma, invasive procedures, lung hyperexpansion, SVC thrombosis, tumours (e.g., lymphoma, teratoma), and infections like tuberculosis and granulomatous diseases [3].

Venous thrombosis is an infrequent cause, but it is an increasingly recognised problem in neonatal care as invasive treatments and procedures become more common. Central vein thrombosis causes a backward pressure increase in the thoracic duct, resulting in the outflow of chyle into the pleural cavity. Preterm neonates, especially those who are critically ill, undergo multiple invasive procedures, such as the use of vascular access devices, which increases their susceptibility to thromboembolic complications alongside other risk factors like sepsis and coagulopathies [4].

In a systematic review, Nossair et al. found that, in the paediatric population, in a subgroup of patients with evidence of SVC thrombus, 45% had ≥2 known thrombotic risk factors, and 87% had a CVC in place [2]. In a neonatal subpopulation, Park et al. reported a 9.2% incidence of thrombosis associated with CVC, with the predominant locations of the thrombus in the hepatic system, right atrium, and superior/inferior vena cava [5].

CVCs play a vital role in managing critically ill neonates, given their prematurity and anticipated long-term nutritional and therapeutic requirements. However, these devices place the child at risk for complications, necessitating ongoing surveillance. In the present case, echocardiographic evaluation revealed the CVC distal tip in an intracardiac position, subjecting it to turbulent blood flow, which may have facilitated thrombosis development at the tip of the CVC, extending into the SVC.

This catheter-related thrombosis was associated with *Staphylococcus capitis* catheter-related sepsis, identified simultaneously in peripheral and catheter blood samples. Over the last two decades, multidrug-resistant *Staphylococcus capitis* has been increasingly reported as a major agent responsible for catheter-related bloodstream infections in preterm neonates [6]. These two factors are commonly associated: the thrombus serves as a nidus for bacterial multiplication, while the CVC surface promotes platelet activation and thrombus formation, whose ligands promote the adherence of local bacteria [7].

Apart from the removal and replacement of the catheter in the right internal jugular vein, we initiated therapy with vancomycin and anticoagulation adjusted to anti-Xa values. After five days, due to the initial lack of thrombus reduction, anticoagulation was changed to UH in perfusion (20 U/kg/h). In addition to the common recommendations for its neonatal use, we considered the limitations described in the literature [8]. Due to the physiological immaturity of the haemostatic system in these infants, with reduced levels of antithrombin resulting in relative resistance to heparin, frequent increases in the administered dose were required due to persistent subtherapeutic values. Therefore, LMWH therapy was reestablished.

Strategies for treating chylothorax include drainage of chyle from the pleural space, reduction in volume output with dietary modifications or medication (octreotide), and maintenance of nutrition, fluid, and electrolyte balance [1].

All of this supportive care had little or no effect on fluid reduction, which aligns with the literature describing high-output chylothorax as often refractory to first-line therapy. This particular type of chylothorax makes patients susceptible to complications due to the significant fluid output that leads to the loss of proteins, electrolytes, fats, and lymphocytes, even with adequate fluid replacement [1]. More invasive interventions are required for refractory cases: surgical intervention or chemical pleurodesis may be needed but are associated with high morbidity and questionable success rates [9]. Thoracic duct ligation has shown significant effectiveness in postoperative chylothorax but is not always possible, especially in patients younger than 4 months or weighing less than 4 kg, such as premature newborns [10].

For the removal of obstructive thrombi, therapeutic options include local and systemic thrombolytic therapy, catheter aspiration thrombectomy, surgery, stent implantation, and recently, stent-retriever thrombectomy [2, 10-12].

The lack of consensus on methods and protocols for SVC obstruction and refractory chylothorax in this age group complicates treatment decisions for critically ill children. Invasive approaches are often delayed until the child deteriorates or death occurs. Criteria for transitioning from conservative to surgical therapy are not standardized. Experience with microsurgery is limited, with only rare reports of thrombectomy in older, heavier children, and the ideal timing of surgery remains undefined.

Surprisingly, the autopsy revealed that the SVC lumen was patent. This finding heightened concerns about the aetiology of the chylothorax. Secondary complications like malnutrition and immunosuppression, despite vessel patency, can be fatal, emphasizing the need for early detection and intervention to reduce mortality. The rising use of central venous lines in neonatal intensive care units predicts more catheter-related thrombosis cases and, therefore, the need for treatment guidelines for chylothorax associated with central venous stenosis/occlusion. Early diagnosis and intervention can hasten recovery, preventing invasive treatments and fatal outcomes.

## Conclusions

This case exemplifies SVC obstruction associated with CVC as a cause of severe high-volume output chylothorax. This is a life-threatening event that demands close monitoring of CVC status and a high index of suspicion for venous obstruction to allow for early intervention. Future efforts should focus on establishing precise criteria to standardize the transition from medical to surgical therapy and optimize current practices.
